# Plasma pharmacokinetics, milk residue depletion profile, and milk withdrawal interval estimation following multiple-dose oral administration of meloxicam to lactating dairy goats

**DOI:** 10.3389/fvets.2025.1620476

**Published:** 2025-07-23

**Authors:** Melissa A. Mercer, Zhicheng Zhang, Maaike O. Clapham, Scott E. Wetzlich, Fauna L. Smith, Benjamin A. Rupchis, Zhoumeng Lin, Lisa A. Tell

**Affiliations:** ^1^Department of Veterinary Medicine and Epidemiology, School of Veterinary Medicine, University of California-Davis, Davis, CA, United States; ^2^Department of Environmental and Global Health, College of Public Health and Health Professions, University of Florida, Gainesville, FL, United States; ^3^Center for Environmental and Human Toxicology, University of Florida, Gainesville, FL, United States; ^4^Center for Pharmacometrics and Systems Pharmacology, University of Florida, Orlando, FL, United States; ^5^Department of Population Health and Reproduction, School of Veterinary Medicine, University of California-Davis, Davis, CA, United States; ^6^Department of Animal Science, University of California-Davis, Davis, CA, United States

**Keywords:** meloxicam, milk withdrawal interval, goats, pharmacokinetics, milk residues, drug residues

## Abstract

**Introduction:**

Meloxicam is frequently administered orally to lactating dairy goats in an extra-label manner. However, since Food and Drug Administration (FDA)-approved withdrawal times have not been established, this raises concerns for potential drug residues in milk. This study aimed to estimate plasma pharmacokinetics, determine meloxicam residues in milk based on concentration versus time depletion profiles, and calculate estimated withdrawal interval (WDI) recommendations for meloxicam following multiple oral doses administered to lactating dairy goats.

**Methods:**

Meloxicam was administered to healthy mid-lactation dairy goats (*n* = 10) at 1 mg/kg orally every 24 h for a total of 6 doses. Meloxicam was quantified in plasma and milk samples using high-performance liquid chromatography (HPLC) with an ultraviolet detector. Plasma pharmacokinetic parameters were estimated using a non-compartmental approach, and theoretical milk elimination half-life was also estimated. Milk WDIs were calculated using the FDA tolerance limit method under various data simulation scenarios and compared to the standard FDA regulatory approach, which involves 10 animals with triplicate samples. Additional assessment included the European Medicines Agency (EMA) maximum residue limit (MRL) method and the theoretical terminal elimination half-life method.

**Results:**

Following the final administered dose, the geometric mean plasma estimated T_1/2_ was 7.64 h (range 5.61–9.47 h), while the geometric mean milk theoretical estimated T_1/2_ was 6.96 h (range 5.47–9.56 h). WDI estimations using the FDA tolerance limit method targeting the analytical limit of detection (4 ng/mL) ranged from 70.1 to 82.8 h. The WDI estimated using the EMA MRL method targeting the EMA MRL (15 ng/mL) was 64.97 h. Monte Carlo simulation of single replicate data closely approximated WDI estimates from full triplicate datasets, whereas simulating additional virtual animals introduced relatively greater variability in the estimated WDI.

**Conclusion:**

This study estimated the plasma pharmacokinetic parameters and theoretical milk residue depletion profiles for meloxicam following multiple oral doses administered to healthy lactating dairy goats. The theoretical elimination half-life of meloxicam for milk is shorter for goats than cattle, resulting in shorter estimated WDIs for the same dosing regimen. From a food safety perspective, meloxicam cattle withdrawal times (WDTs) or estimated WDIs may be appropriately conservative for application to goats when properly adjusted for dose, route, and regulatory tolerances/MRLs.

## 1 Introduction

While still considered a minor food animal species, the total number of goats in the United States (US) is estimated to be over 2.5 million, and a 4% annual increase was observed in the number of dairy goats in the US between 2024 and 2025 (1). Like other ruminant species, goats commonly require analgesia and anti-inflammatory therapy to alleviate the discomfort and inflammation associated with disease and/or surgical procedures, including disbudding, castration, and cesarean section (2–5). However, despite the increasing population of goats and welfare considerations associated with pain and inflammation, there are currently no anti-inflammatory or analgesic drugs approved by the US Food and Drug Administration (FDA) for use in any small ruminant species in the US. Therefore, all analgesic or anti-inflammatory drug use in goats in the US is considered extra-label drug use (ELDU) and is thus subject to regulation under the Animal Medicinal Drug Use Clarification Act of 1994 (AMDUCA) and US Code of Federal Regulations 21 CFR 530 (6, 7). Under these same regulations, for any ELDU in food-producing animals in the US, a greatly extended withdrawal interval (WDI) must be issued by the prescribing veterinarian to protect human food safety and ensure that prohibited drug residues do not occur in the food products.

There are several approaches for estimating ELDU WDIs. The simplest method is to leverage the assumption that more than 99% of a drug is depleted from a tissue after 10 elimination half-lives (8, 9). Therefore, in simple words, the terminal elimination half-life method multiplies the terminal tissue or product half-life by a factor of 10 (8, 9). The regulatory approaches, however, rely on statistical methods to establish a withdrawal period for the 95th percentile of population (European Medicines Agency, EMA) (10) or the 99th percentile of the population (U.S. Food and Drug Administration, U.S. FDA) (11) with a 95% confidence interval (CI) using software applications. As these regulatory methods base their withdrawal periods on a representative range of a population mean compared to the sample mean used in the terminal elimination half-life approach, they have the advantage of providing a more conservative ELDU WDI estimate that considers a wider population variance. However, the regulatory methods assume a homogenous population of animals and a normally distributed dataset derived from a good laboratory practice (GLP) study, which is not always feasible in small studies funded to examine residue depletion following ELDU in food animals. Therefore, for some studies where the number of animals sampled or the number of samples procured per time point are insufficient based on the minimum requirements of the regulatory approaches, Monte Carlo simulation is performed to generate additional in silico animals or samples based on the mean sample concentration and population coefficient of variation or standard deviation (SD) (9, 12–14). However, it is unknown how closely Monte Carlo simulation mirrors the WDI estimations produced by studies that fit the minimum requirements of the regulatory approaches, and what scenarios (such as less animals than the minimum requirements, less samples collected, and reduced number of replicates analyzed per sample) may be best suited for generating in silico datapoints.

Meloxicam (4-hydroxy-2-methyl-N-(5-methyl-2-thiazolyl)-2H-1,2-benzothiazine-3-carboxamide-1,1-dioxide) is a non-steroidal anti-inflammatory drug (NSAID) of the oxicam class group that preferentially inhibits cyclooxygenase-2 (COX-2) and therefore reduces the production of pro-inflammatory prostaglandins. In contrast to non-selective COX-inhibitors, such as flunixin meglumine or phenylbutazone, meloxicam appears to have a more favorable safety profile, leading to increasing interest in its use in ruminant species (15). Furthermore, flunixin meglumine has been associated with an increased risk of retained fetal membranes and metritis in postpartum cattle (16, 17). However, meloxicam does not carry this risk, which makes it a more attractive analgesic option for use in postpartum ruminants (18).

A query of internal submission data from the US Food Animal Residue Avoidance Databank (FARAD) program found that the most common dose, route, frequency, and duration of dosing for meloxicam administered to goats was 0.5–1 mg/kg orally every 24 h for 5–7 days of treatment, with 49.5% of submissions requesting milk WDI recommendations (19). The plasma pharmacokinetics of single-dose meloxicam in goats has been extensively documented for intravenous (IV), intramuscular (IM), oral, and subcutaneous routes (2, 20–23). However, no studies have reported the plasma kinetics for multiple dose administration of meloxicam to goats via any route (24). Additionally, meloxicam milk residue depletion and WDI estimation have been reported for single-dose IV or IM administration in goats, which suggested a 68 and 64 h milk withdrawal for 0.5 mg/kg single-dose IV and IM administration, respectively (20, 23). However, it should be noted that the calculated milk WDI recommendations for a 0.5 mg/kg single-dose IV or IM administration in goats was based on the EMA method targeting the EMA bovine/caprine milk tolerance of 15 ppb (23). In the US, since meloxicam is not currently approved for use in food producing species, the milk tolerance for residues is zero; therefore, the WDI applied by a veterinarian following ELDU must ensure that milk residues are below the most sensitive regulatory analytical limit of detection (LOD). Since there is no listed method for meloxicam milk residue detection in the FDA’s Milk Guidance Documents & Regulatory Information System (25) or the National Milk Drug Residue Database (NMDRD) (26), the analytical method’s LOD serves as a proxy tolerance when estimating WDIs for meloxicam in milk in the US.

The objectives of this study were to estimate the plasma pharmacokinetic parameters, visualize the milk residue depletion profile, and calculate milk WDI estimates using multiple approaches based on time versus concentration data obtained following oral administration of meloxicam to lactating dairy goats at 1 mg/kg every 24 h for a total of 6 doses. Additionally, this study aimed to determine the validity of the Monte Carlo simulation to generate additional in silico animals or datapoints to meet the minimum requirements of regulatory methods to provide WDI estimations. Finally, these results were compared to estimated WDIs for dairy cattle from a previously performed study (27).

## 2 Methods

### 2.1 Animals

Goats were eligible for enrollment if they were systemically healthy and had no history of meloxicam, NSAID, or avermectin treatment in the previous 30 days. The goats were evaluated prior to enrollment based on physical examination (temperature, pulse, respiration rate, rumen contractions, body condition score, FAffa MAlan CHArt (FAMACHA) test, and udder palpation), treatment history, milk production, and milk somatic cell count. Based on pre-enrollment evaluation, 10 healthy, early to mid-lactation dairy goats in the age range of 2 to 4 years (mean 2.9 ± 0.7 years) and body weight ranging from 67.5 to 102.2 kg (mean 85.8 ± 9.7 kg) were included in the study. The selected breeds included Alpine (*n* = 5), Saanen (*n* = 4), and Saanen/Alpine Cross (*n* = 1). The goats were an average of 70.8 ± 12.6 days in milk.

All goats were housed in group pens (*n* = 10/pen) at the University of California, Davis Animal Science Goat Teaching and Research Facility. Goats were fed alfalfa hay ad libitum and had unrestricted access to fresh drinking water throughout the study. After completion of the sampling protocols, all remaining goats were returned to the facility herd. All procedures relating to this study were performed in strict accordance with protocol #22818 approved by the Institutional Animal Care and Use Committee of the University of California at Davis.

### 2.2 Drug administration

Goats were administered 1 mg/kg meloxicam (Meloxicam tablet 15 mg, Cipla USA, Inc.) via oral drench syringe every 24 h for a total of 6 doses. The dose was rounded to the nearest whole tablet, with an actual mean dose of 1.0 mg/kg (range 0.92–1.11 mg/kg). The dosage regimen and drug formulation (tablets) selected for this study were based on FARAD internal submission data for meloxicam in goats, which mirror the dosing regimen and drug formulation used in a previous cattle study, allowing for comparison of meloxicam kinetics following multiple oral dose administration between goats and cattle (27). The tablets were dissolved in 20 mL of water for initial drug administration, and then 20 mL of water was administered orally to flush any remaining drug residue in the syringe. Syringes were carefully examined post-dosing to ensure all of the drug had been administered. Goats were weighed at the start of the initial dose and again at the end of the study (post-study mean 87.1 ± 9.7 kg). The meloxicam dose was recalculated to ensure accuracy with the pre-assigned dosage regimes, with a calculated post-study average administered dose of 0.98 ± 0.06 mg/kg.

### 2.3 Plasma and milk collection

Blood was collected via direct venipuncture of the jugular vein into 10 mL sodium heparin blood tubes using a vacutainer and a 20 gauge needle apparatus. The blood samples were obtained immediately before the first administered dose of meloxicam (*t* = 0 h) and then subsequently at 1, 2, 4, 8, 12, and 24 h after the first administered dose. Blood sample collection was repeated immediately before the final (6th) administered dose (120 h following the first dose) and then subsequently at 121, 122, 124, 128, 132, 144, 156, 168, 192, 216, 240, 264, and 288 h after the first dose. Equivalent sampling times were 1, 2, 4, 8, 12, 24, 36, 48, 60, 96, 144, and 168 h post-last dose. Blood tubes were placed on ice immediately following sample collection and centrifuged at 2730 x *g* for 10 min at 21°C. Plasma samples were manually harvested and transferred to storage tubes, which were then immediately frozen and stored at −70°C until analysis.

Milk samples were obtained from the goats every 12 h until 456 h post-first dose (12 days of milk collection at 12 h intervals post-last dose). A 0.5% iodine teat-dipping solution was used for pre- and post-dipping of teats. After application, the teats were dried with paper towels after a minimum contact time of 40 s. Prior to milk collection, each teat was stripped twice, and fore-milk was examined for abnormal milk. Milk was collected from each goat using proportionate milk sampling device, which provided a representative sample of all milk produced by each half during the milking process. A total of 50 mL of milk was collected from each goat at each milking, and the remaining milk produced by each goat was diverted and not added to the bulk tank to prevent any potential drug residues from entering the human food chain. The volume of milk produced at each milking by each goat was quantified at the time of sample collection, and every milk sample was immediately frozen and stored at −70°C until analysis. Plasma samples were stored for a maximum of 11 days prior to analysis, while milk samples were stored for a maximum of 52 days, both of which are considered acceptable based on a previous meloxicam stability study performed in goat milk and plasma (20).

### 2.4 Drug analysis

Meloxicam standard was obtained from European Pharmacopoeia Reference Standard (EDQM, Strasbourg, France). Piroxicam (Alfa Aesar, Ward Hill, MA, USA) was used as the internal standard. High-performance liquid chromatography (HPLC)-grade methanol and acetonitrile, dimethyl sulfoxide, potassium phosphate monobasic, phosphoric acid, and sodium sulfate were obtained from Fisher Scientific (Fair Lawn, NJ, USA). Purified water was obtained using a Nanopure water system (Barnstead, Dubuque, IA, USA).

Plasma and milk meloxicam concentrations were quantified using an HPLC with an ultraviolet detector. The HPLC system consisted of an Alliance 2,695 separations module and a 2,487 dual wavelength absorbance detector, and separation was achieved on a Nova-Pak C18, 4-μm, and 300 × 3.9 mm column (Waters, Milford, MA, USA). Chromatographic conditions and preparation of standards and quality control samples were adapted from Depenbrock et al., 2021 (28). The column temperature was maintained at 30°C, and the samples were kept at 10°C. The isocratic mobile phase was 50:50 with 50 mM potassium phosphate buffer (pH 2.15) and acetonitrile set at a flow rate of 0.8 mL/min. Injection volume was 50 μL. Peaks were detected at a wavelength of 355 nm, and the total run time was 10 min.

A primary stock solution of meloxicam (1.0 mg/mL) was prepared in dimethyl sulfoxide. This was diluted to a secondary stock solution (0.1 mg/mL) in 50% methanol (1:1 methanol to water) weekly. The secondary stock solution was used to create a series of working standard solutions (50 to 5,000 ng/mL), also in 50% methanol, which were prepared fresh for each analysis. A primary stock solution of piroxicam (1.0 mg/mL) in dimethyl sulfoxide and a secondary stock solution (0.1 mg/mL) in 50% methanol were similarly prepared. A 500 ng/mL working solution in 50% methanol was diluted from the secondary stock solution. Equal volumes of the meloxicam working solutions and the internal standard working solution were mixed for the standard curve (10–1,000 ng/mL for plasma and milk). Three different concentrations of quality control samples (20, 100, and 400 ng/mL) were prepared in a control matrix (plasma, milk) with each analysis, along with a matrix blank. Control plasma and milk were harvested from venous blood and milk collected prior to study initiation, respectively. The lower limit of quantification (LLOQ) for milk and plasma was 15 ng/mL, and the limit of detection (LOD) was 4 ng/mL.

Plasma and milk samples (250 μL) were spiked with 50 μL of the working internal standard solution prior to the addition of 3.5 mL of acetonitrile. After vortex mixing, the samples were centrifuged at 2730 × g for 10 min. The extractant was transferred to a new tube and evaporated to dryness at 60°C with a gentle stream of nitrogen, then reconstituted with 100 μL of 50% methanol, and centrifuged at 12,000 × g for 3 min before analysis on the HPLC system. Individual milk samples were analyzed in triplicate.

### 2.5 Method validation

Plasma and milk matrices were validated according to the FDA Bioanalytical Method Validation Guidance for Industry (29). Five replicates at each concentration were calculated on a single day for intra-day precision, and five replicates at each concentration were calculated over three consecutive days for inter-day precision. The ratio of meloxicam to the internal standard peak areas with 1/X^2^ weighting was created for calibration curves. The LOD was calculated using blank quality controls for each matrix analyzed in each sample set, with three times the standard deviation of baseline measurements added. The lower limit of quantification (LLOQ) was measured as at least five times the standard deviation of the baseline measurement according to the FDA Guidance for Industry (29).

### 2.6 Pharmacokinetic analysis

Non-compartmental pharmacokinetic analysis of plasma time versus concentration data was performed using concentration–time data collected from each animal. The relevant estimated pharmacokinetic parameters were summarized as geometric means (GMs), geometric standard deviations (GSDs), and ranges. All analyses were performed using a commercial software program (Phoenix WinNonlin 8.4.0.6172, Certara, Princeton, NJ, USA) with a non-compartmental pharmacokinetic analysis (NCA) approach. For plasma, the estimated pharmacokinetic parameters defined below were reported:

- C_max (obs)_: Maximum observed concentration, occurring at time $T_{\max.}$
- T_max (obs):_ Time of maximum observed concentration
- T_1/2 (elim)_: Terminal elimination half-life using the ratio of the natural log of 2 and λz
- λz: Terminal elimination rate constant using a linear regression of the terminal log-linear portion of the plasma meloxicam concentration profile
- Vd/F: Volume of distribution per fraction of dose absorbed for the terminal phase
- CL/F: Total body clearance per fraction of dose absorbed as the ratio between dose and AUC_0-∞_
- AUC_0_-*_τ_*: The area under the concentration–time curve from time 0 to τ (24) h after administration using the linear trapezoidal method
- AUC_0-∞:_ The area under the concentration–time curve from the time of the first dose (0 h) extrapolated to infinite time using the linear trapezoidal method
- AUC_120-∞:_ The area under the concentration–time curve from the time of the last dose (120 h) extrapolated to infinite time using the linear trapezoidal method
- AUC_extrapolated: Percentage of extrapolated area of AUC_0-∞_ from the time of the last measured concentration to infinity using the linear trapezoidal method
- MRT_0-∞_: Mean residence time extrapolated to infinity
- R_ac_: Accumulation ratio indicates how much a drug will accumulate in the body after multiple doses, compared to a single dose. It is calculated as $\text{AU}C_{\left(0-\tau ,\text{ss}\right)}/$$\text{AU}C_{\left(0-\tau ,1\right)}.$

For the meloxicam milk analysis, since milk is an excretory product, two specific pharmacokinetic parameters were directly observed from the data (observed maximum time and concentrations) and two theoretical pharmacokinetic parameters were estimated individually for each goat using a non-compartmental approach with commercial software, namely, observed maximum concentration (C_max (obs)_), observed time of maximum concentration (T_max (obs)_), theoretical terminal elimination half-life (T_1/2 (elim theo)_), and theoretical area under the curve extrapolated to infinity (AUC_120-∞ (theo)_).

### 2.7 Withdrawal interval estimation

Milk WDIs were estimated using three pharmacostatistical methods, including the terminal elimination half-life approach (8), the tolerance limit method from the U.S. FDA (11), and the method from the European Medicines Agency (EMA) (10). The terminal elimination half-life method was performed as described earlier, whereby the terminal elimination half-life for plasma or each tissue was multiplied by a factor of 10 to serve as an estimate of the WDI (8). Different standards were used as operational tolerances in the FDA and EMA methods. For the FDA method, the plasma and milk WDI for meloxicam were estimated via the US FDA tolerance limit method in the “reschem” R package (11). The WDI was estimated as the interval during which the 99th percentile tolerance limit on the residue concentration was at or below tolerance with a 95% confidence level. Since meloxicam is not approved for use in any food-producing species in the US, there is zero tolerance for milk and tissue residues. We, therefore, applied the limit of detection (LOD) of 4 ng/mL of the analytical method used in this study as the operational tolerance.

Despite fulfilling the FDA method’s minimum requirement of at least 10 animals with triplicate samples per time point, due to the 12 h sampling scheme and rapid elimination of meloxicam from milk, our milk dataset did not have four terminal elimination phase time points available for all 10 animals. Specifically, goats 5, 6, 7, 9, and 10 had only three terminal phase time points when the meloxicam concentrations were quantifiable. To address this limitation and to evaluate the robustness of simulated in silico datapoints generated in pharmacokinetic studies where complete datasets are unavailable, we proposed three scenarios (Table 1) based on various situations from previously published residue depletion studies (9, 12, 13).

**Table 1:** Analytical scenarios for extra-label withdrawal interval (WDI) estimation for meloxicam in goat milk using the FDA method.

Scenario	Description	Methodology
Scenario 1: actual triplicate data with minor adjustments	Utilizes actual triplicate milk concentration data from 10 animals. However, some animals do not meet the FDA’s requirement for at least four terminal-phase time points.	For study goats (goats 5, 6, 7, 9, and 10) that had fewer than four time points in the terminal elimination phase, the fourth time point (study time 167 h after the last drug dose administered) was assigned a concentration equal to 4 ng/mL (LOD).
Scenario 2: virtual animal data simulation using data from 5 study animals for generating simulated data	Mimic scenarios where limited animal data prevent meeting the FDA’s requirement of 10 animal subjects.	1. Data from study animals (goats 1, 2, 3, 4, and 8) were used to generate meloxicam concentration values for five additional virtual animals. 2. The meloxicam concentration values for the simulated animals were derived from the mean and coefficient of variation calculated from actual study data from five study animals at each time point.
Scenario 3: use of a single milk concentration value (Replicate A) to simulate data in triplicate	Mimic scenarios where a sample is only analyzed once for each time point, yet triplicate data are required for the FDA mild tolerance limit method.	1. A single residue concentration value (Replicate A) for all sampling time points for each study animal served as the baseline for simulating two additional data points per time point for all study animals. 2. The two additional replicate residue concentration values per sample time point were generated for each study animal using the pooled mean and coefficient of variation derived from Replicate A.

In Scenario 1, real triplicate data from all 10 goats were used in accordance with FDA guidelines; however, for those five goats lacking a fourth terminal phase time point, a concentration of 4 ng/mL (the analytical method’s limit of detection) was assigned to the 167 h sampling time point. Scenario 2 was designed to handle cases of insufficient animal numbers by employing virtual animal simulation based on previous studies (12, 13) where virtual replicates were used to expand a dataset from five to ten animals. Specifically, in Scenario 2, five goats (Goats 1, 2, 3, 4, and 8) with at least four terminal elimination phase time points were used to generate five additional in silico goats, with the simulated milk concentration values derived from the mean and coefficient of variation for the five baseline goats at each time point. Lastly, Scenario 3 mimics conditions in which only a single measurement is available per time point for each animal (9, 13, 14). A single measured concentration for each goat at each time point (the first replicate (Rep) A) served as the baseline. From this single replicate dataset, two additional replicates (“simulated_repB” and “simulated_repC”) were generated by applying the pooled mean and coefficient of variation derived from Rep A. By comparing the results across these three scenarios, we aimed to evaluate the reliability and predictive accuracy of virtual data generation within the FDA’s tolerance limit framework. This comparative approach provides insights into the robustness of the “reschem” R package and its applicability for handling real-world challenges in estimating WDI when experimental data are insufficient.

For the EMA method, the plasma and milk WDIs were calculated using the EMA’s WTM 1.4 software program based on the EMA’s maximum residue limit (MRL) method. The calculated WDI represents the time when the upper one-sided 95% tolerance for the residue is below the MRL level with 95% confidence, meaning that 95% of animals’ tissue/milk drug concentrations will be under the MRL level with 95% confidence (10). MRLs for meloxicam have been established in the European Union (EU) for cattle milk at 15 ng/mL, and the EU has also extended MRLs to include goats. Thus, it was assumed that the MRLs set for cattle are sufficiently conservative for use in the present analysis of goats’ plasma and milk data (30).

## 3 Results

All goats remained clinically healthy over the entire study period, with no adverse effects noted during or following meloxicam administration. The LOD, LLOQ, precision, accuracy, intra-assay variation, internal standard recovery, and meloxicam recovery for plasma and milk samples are presented in Table 2. The calibration curve was linear from 10 ng/mL to 1,000 ng/mL with the average R-squared value of 0.9946. Selectivity was demonstrated by analysis of blank samples from six individual sources for both plasma and milk, and neither showed interfering peaks at the retention times for meloxicam or the internal standard. Freeze–thaw stability testing was not performed, as all samples were only thawed once immediately prior to analysis.

**Table 2:** Sensitivity, precision, and accuracy parameters for the high-performance liquid chromatography analytical method used to measure meloxicam concentrations in plasma and milk following oral meloxicam administration in goats.

Parameter	Plasma	Milk
LOD (ng/mL)	4	4
LLOQ (ng/mL)	15	15
Precision (%)	2.4	3.3
Accuracy (%)	101.4	104.0
Intra-Assay Variation (%)	2.0	1.9
Internal Standard Recovery (%)	85.5	86.7
Meloxicam Recovery (%)	87.7	91.3

LOD, Limit of detection; LLOQ, Lower limit of quantification.

**Figure 1:**
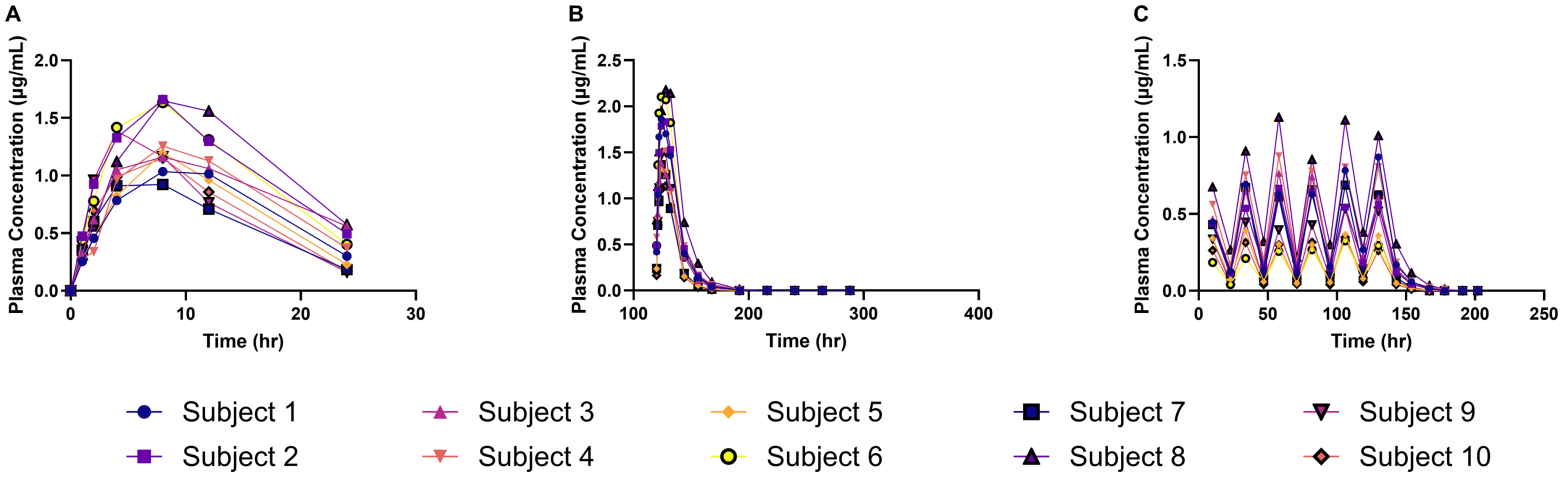
Meloxicam concentration vs. time curves for plasma and milk samples collected from lactating dairy goats (*n* = 10) following oral administration of meloxicam at 1 mg/kg every 24 h for a total of 6 doses. **(A)** The plasma concentration of meloxicam and the time on the x-axis represents time points after the first dose; **(B)** The plasma concentration of meloxicam and the time on the x-axis represents time points after the last dose; **(C)** The milk concentration of meloxicam and the time on the x-axis represents time points after the first dose.

The meloxicam concentration–time profiles obtained from plasma and milk samples following repeated daily oral administration of 1 mg/kg meloxicam for 6 days in goats are shown in Figure 1. In Figure 1A, meloxicam plasma concentrations are plotted against time after the first dose, while Figure 1B depicts the plasma concentration–time curve after the final (sixth) dose. Figure 1C demonstrates meloxicam concentrations measured in milk, with sampling times expressed relative to the first dose.

Following the first administered dose of meloxicam at 1 mg/kg orally to lactating dairy goats, the geometric mean plasma C_max(obs)_ was 1.28 μg/mL (range 0.92–1.66 μg/mL), with a geometric mean plasma T_max(obs)_ of 7.46 h (range 4–8 h), and a geometric mean estimated plasma T_1/2 (elim)_ of 7.49 h (range 5.45–14.37 h). The geometric mean estimated AUC_0_ *τ* was 19.86 h^*^μg/mL (range 14.46–27.03 h^*^μg/mL), the geometric mean estimated AUC_0-∞_ was 23.73 h*μg/mL (range 16.20–33.91 h*μg/mL), and the estimated AUC_0-∞_ was 14.13% (range 6.77–35.77%) extrapolated due to the shortened sampling window (0- *τ*) following the first administered dose.

Following the final administered dose of meloxicam at 1 mg/kg orally every 24 h for a total of 6 doses to lactating dairy goats, the geometric mean plasma C_max (obs)_ was 1.59 μg/mL (range 1.14–2.18 μg/mL) with a geometric mean plasma T_max(obs)_ of 5.66 h (range 4–8 h) and a geometric mean plasma estimated T_1/2(elim)_ of 7.64 h (range 5.61–9.47 h). The geometric mean estimated AUC_0_ *τ* was 26.28 h^*^μg/mL (range 18.27–39.91 h^*^μg/mL), the geometric mean estimated AUC_0-∞_ was 30.10 h*μg/mL (range 19.66–49.87 h*μg/mL), and the estimated AUC_0-∞_ was 0.33% (range 0.17–0.67%) extrapolated. The plasma accumulation ratio (R_ac_) for meloxicam was calculated to be a geometric mean of 1.32 (range 1.08–1.74). The remainder of the estimated plasma pharmacokinetic parameters are reported in Table 3. Given the lack of comparable IV dosing, limited estimated pharmacokinetic parameters are reported.

**Table 3:** Estimated non-compartmental pharmacokinetic parameters from plasma meloxicam concentration vs. time data following oral administration of meloxicam to lactating dairy goats (*n* = 10) at 1 mg/kg every 24 h for a total of 6 doses.

Parameter	Dosing regimen					
	After first dose			After last dose		
	Geometric mean	GSD	Range	Geometric mean	GSD	Range
$C_{\max(\text{obs})}$ (μg/mL)	1.28	1.23	0.92–1.66	1.59	1.24	1.14–2.18
$T_{\max(\text{obs})}$ (h)	7.46	1.25	4–8	5.66	1.44	4–8
$T_{1/2(\text{elim})}$ (h)	7.49	1.33	5.45–14.37	7.64	1.20	5.61–9.47
λz (1/h)	0.093	1.33	0.048–0.13	0.091	1.20	0.07–0.12
Vd/F (mL/kg)	455.30	1.21	353.7–637.4	366.20	1.24	243.2–460.4
CL/F (mL/kg/h)	42.14	1.32	29.49–61.71	33.22	1.36	20.05–50.86
$\text{AU}C_{0-\tau }$ (h*μg/mL)	19.86	1.24	14.46–27.03	26.28	1.30	18.27–39.91
$\text{AU}C_{0-\infty }$ (h*μg/mL)	23.73	1.32	16.20–33.91	----	----	----
$\text{AU}C_{120-\infty }$ (h*μg/mL)	----	----	----	30.10	1.36	19.66–49.87
$\text{AU}C_{0-\infty \text{or}\;120-\infty }$ extrapolation (%)	14.13	1.66	6.77–35.77	0.33	0.17	0.17–0.67
MRT_0-∞_ (h)	14.04	1.26	10.24–23.21	12.13	1.15	10.33–15.03
$R_{\text{ac}}$	----	----	----	1.32	1.15	1.08–1.74

GSD: Geometric standard deviation. The following individual parameter estimates are reported: C_max(obs)_: maximum observed plasma concentration; T_max(obs)_: time to maximum observed plasma concentration; T_½(elim)_: elimination half-life based on best fit data points; λz: elimination rate constant based on best fit data points; Vd/F: apparent volume of distribution during the terminal elimination phase; CL/F: apparent total clearance of drug from plasma; $({\text{AUC}}_{0-\tau }):$ area under the plasma concentration vs. time curve to the next dosing interval (24 h); $({\text{AUC}}_{0-\infty }):$ area under the plasma concentration vs. time curve from the time of the first dose extrapolated to infinity; $({\text{AUC}}_{120-\infty }):$ area under the plasma concentration vs. time curve from the time of the last dose extrapolated to infinity; AUC_(0-∞ or 120-∞)_ extrapolation (%): percent of the ${\text{AUC}}_{0-\infty }$ following the first dose, or $\;{\text{AUC}}_{120-\infty }$ following the final administered dose, that was extrapolated; MRT_0-∞_: mean residence time, based on the plasma concentration–time curve extrapolated to infinity; R_ac_: accumulation ratio calculated as AUC_((0-τ,ss))_/AUC_((0-τ,1))_.

**Figure 2:**
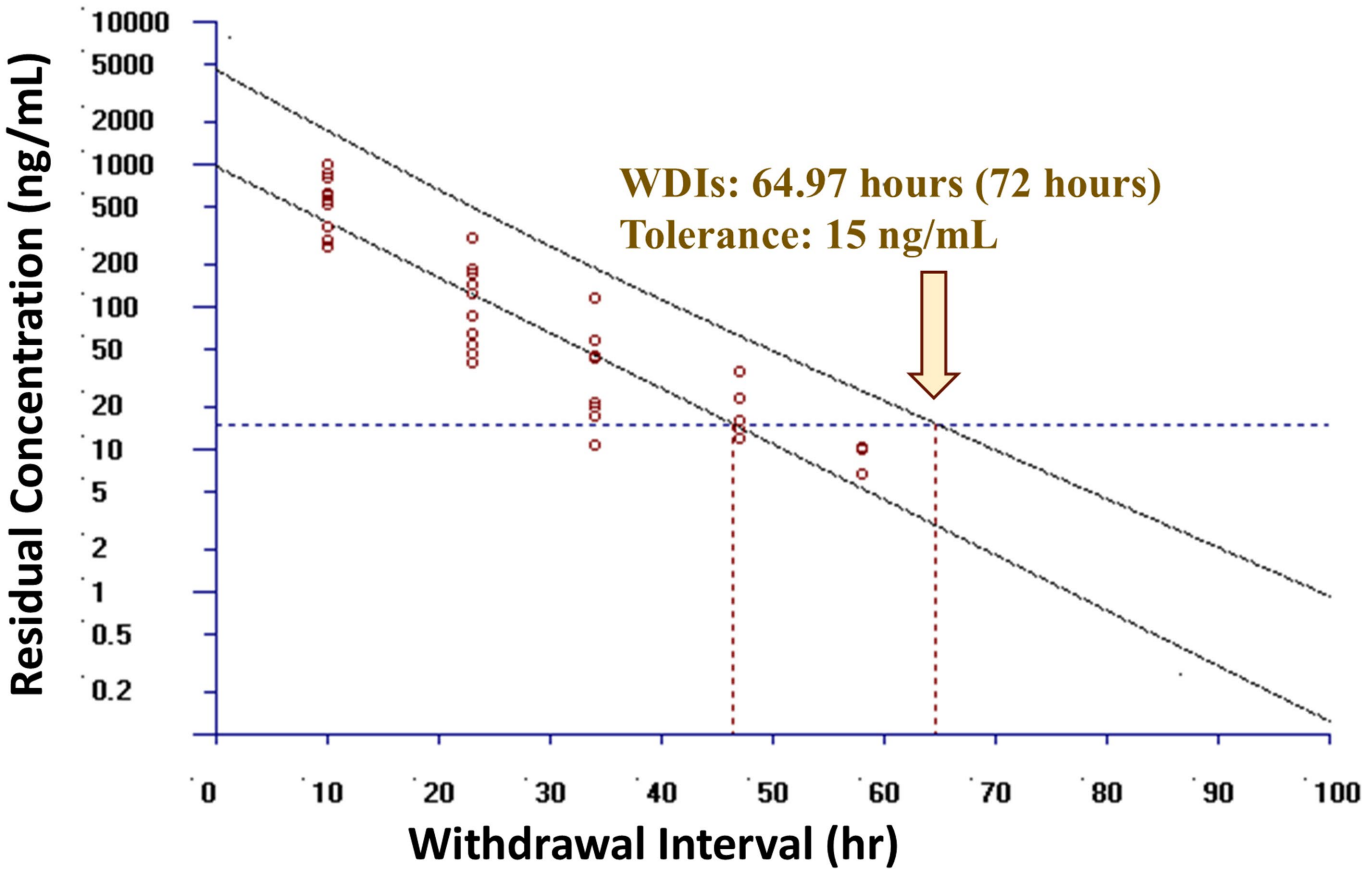
Estimated post-last dose milk withdrawal interval (WDI) using the European Medicines Agency (EMA) maximum residue limit (MRL) method for lactating dairy goats (*n* = 10) following oral administration of meloxicam dosed at 1 mg/kg every 24 h for a total of 6 doses. The maximum residue limit value used was the EMA MRL of 15 ng/mL in goat milk. Open circles represent actual data points included in the models. Yellow arrow represents the estimated WDI. If the estimated WDI was a fraction, in practice it would be rounded up to the next 12 h interval based on a 12 h milking interval, as shown in the bracket.

Observed and theoretical estimated milk pharmacokinetic parameters are presented in Table 4. For milk, the geometric mean C_max(obs)_ following the final (6th) administered dose of meloxicam at 1 mg/kg orally was 0.54 μg/mL (range 0.30–1.00 μg/mL), and the T_max(obs)_ was 10 h for all 10 goats. The geometric mean estimated milk T_1/2(elim theo)_ was 6.96 h (range 5.47–9.56 h). The estimated ELDU WDIs for milk following oral administration of meloxicam at 1 mg/kg orally ever 24 h for a total of 6 doses to lactating goats using the theoretical milk terminal elimination half-life method, the FDA tolerance limit method (for Scenarios 1–3), and the EMA MRL method are presented in Table 5. The longest estimated milk WDI was 82.8 h (rounded to 84 h based on 12 h milking intervals). This WDI was calculated using the FDA tolerance limit method to target a tolerance of the analytical LOD (4 ng/mL) for Scenario 2, where 5 animals have triplicate sampling, and a Monte Carlo simulation was used to generate 5 “in silico” animals to fulfill the minimum requirements for the calculation method. The shortest estimated milk WDI was 64.97 h (rounded to 72 h based on 12 h milking intervals), which was calculated using the EMA MRL method targeting the EMA MRL of 15 ng/mL (Figure 2). The terminal elimination half-life method using the estimated milk T_1/2(elim theo)_ yielded an intermediate WDI (69.6 h rounded to 72 h), as did the FDA tolerance limit method using the analytical LOD for Scenario 1 (70.1 h rounded to 72 h) and Scenario 3 (70.3 h rounded to 72 h).

**Table 4:** Non-compartmental pharmacokinetic parameters estimated from milk meloxicam concentration versus time data following oral administration of meloxicam to lactating dairy goats (*n* = 10) at 1 mg/kg every 24 h for a total of 6 doses.

Estimated pharmacokinetic parameter	Goat #1	Goat #2	Goat #3	Goat #4	Goat #5	Goat #6	Goat #7	Goat #8	Goat #9	Goat #10	Geometric mean	GSD
$C_{\max(\text{obs})}$ (μg/mL)	0.87	0.56	0.60	0.81	0.36	0.30	0.62	1.00	0.52	0.26	0.54	0.24
$T_{\max(\text{obs})}$ (h)	10	10	10	10	10	10	10	10	10	10	10.0	0
$T_{1/2(\text{elim theo})}$ (h)	6.85	7.05	9.56	9.34	7.26	6.57	5.47	6.49	6.34	5.77	6.96	1.30
$\text{AU}C_{120-\infty \text{milk}\;(\text{theo})}$ (h*μg/mL)	13.01	8.64	9.49	12.66	4.98	4.30	8.49	17.21	7.06	3.65	8.01	4.10
$\frac{\text{AU}C_{120-\infty \text{milk}\;(\text{theo})}}{\text{AU}C_{120-\infty (\text{plasma})}}$ (h*μg/mL)	0.37	0.23	0.35	0.36	0.21	0.11	0.39	0.35	0.29	0.19	0.27	1.51

GSD: Geometric standard deviation. The following individual parameter estimates are reported: Cmax(obs): maximum observed milk concentration; Tmax(obs): time to maximum observed milk concentration; T_½(elim theo)_: theoretical milk elimination half-life based on best fit data points; AUC_(120-∞ (theo))_: area under the theoretical milk concentration vs. time curve from the time of the last dose extrapolated to infinity; $\frac{{\text{AUC}}_{120-\infty \text{milk}\;(\text{theo})}}{{\text{AUC}}_{120-\infty (\text{plasma})}}:$ milk to plasma ratio (described by AUC_120-∞ milk(theo)/_ AUC_120-∞ plasma_) used to determine drug penetration into milk.

**Table 5:** Estimated extra-label withdrawal intervals (WDI) for meloxicam in goat milk following oral administration to lactating dairy goats (*n* = 10) at 1 mg/kg every 24 h for 6 doses.

Method	Tolerance/MRL	Milk (h) (Rounded to next milking interval)
Terminal theoretical milk elimination half-life method	Theoretical 99% drug depletion	**69.6** (72)
FDA tolerance limit method (Scenario 1)	4 ng/mL	**70.1** (72)
FDA tolerance limit method (Scenario 2)	4 ng/mL	**82.8** (84)
FDA tolerance limit method (Scenario 3)	4 ng/mL	**70.3** (72)
EMA maximum residue limit method	15 ng/mL	**64.97** (72)

The terminal elimination half-life method refers to the principle that 99% of drug depletion occurs following 10 half-lives. Therefore, for this method, the milk theoretical terminal elimination half-life is multiplied by a factor of 10. The EMA method refers to the computerized version of the method (WTM 1.4 software) used to calculate withdrawal periods for milk, developed by the Committee for Veterinary Medicinal Products (CVMP) of the European Medicines Agency (EMA). The FDA method refers to the tolerance limit method (coded in the “reschem” R package) used to calculate milk withdrawal times, developed by the U.S. Food and Drug Administration (FDA). Scenario 1 refers to 10 goats with triplicate milk samples, where, when fewer than four terminal elimination timepoints have a value <limit of detection (LOD), the LOD (4 ng/mL) is assigned to those timepoints, so every animal has at least four terminal elimination timepoints. Scenario 2 refers to a scenario where 5 animals have triplicate sampling, and the Monte Carlo simulation is used to generate 5 “in silico” animals to fulfill the minimum requirements for the calculation method. Scenario 3 refers to a scenario where 10 animals have single replicate sampling, and the Monte Carlo simulation is used to generate additional replicates, so that all 10 animals have triplicate sampling. Both FDA and EMA methods output the exact time the drug concentration is below the tolerance or maximum residue limit (shown in bold), and this time is rounded up to the next milking interval based on a 12 h milking schedule (shown in parentheses).

## 4 Discussion

This study estimated the meloxicam pharmacokinetic parameters and milk residue depletion in early to mid-lactation dairy goats following repeated oral dosing and compared these findings to existing data for single-dose administration in goats and multiple-dose administration in lactating dairy cattle (27). Our results demonstrated that lactating dairy goats administered multiple doses of meloxicam have slightly shorter estimated plasma and theoretical milk elimination kinetics compared to lactating dairy cattle under the same dosing regimen, suggesting faster systemic and milk clearance of meloxicam in goats (27). Consequently, all calculated milk WDIs for goats were shorter than those proposed for cattle, indicating that cattle-derived WDIs are likely conservative when applied to lactating dairy goats with the same dosing regimen and the same regulatory tolerance/MRL (27). However, it should be noted that this study administered meloxicam tablets dissolved in water via oral drench, while the cattle study administered whole tablets in gelatin capsules. Therefore, the differences in administration methods may have resulted in more rapid absorption of meloxicam in this goat study. This study also evaluated two Monte Carlo simulation strategies for generating additional replicate or virtual animal data to support regulatory WDI estimation when complete datasets are unavailable. Simulating additional replicates from single replicate data yielded WDI estimates nearly identical to those based on full triplicate datasets, while simulating additional animals introduced modest variability that extended the estimated WDI by 12 h. Therefore, simulation of triplicate data using single replicate data may be permissible to generate a WDI from studies where only single replicate data are available. These findings show that simulation of triplicate data from single-replicate studies may provide a cost-effective means of establishing a WDI following ELDU in minor species. The findings of this study have important implications for establishing and extrapolating evidence-based WDIs in goats. The findings also show that the application of cattle WDIs or Withdrawal Times (WDTs) to goats, when properly adjusted for tolerance or MRL, can be appropriately conservative from both a regulatory and food safety standpoint. However, these findings also emphasize the need for developing disease-specific and model-informed approaches in future work.

The observed differences in estimated plasma pharmacokinetic parameters between multiple-dose administration in this study and single-dose administration in previous studies are relatively modest and likely due to differences in dose, lactation status, and product formulation. The geometric mean plasma C_max(obs)_ following the first administered oral dose of meloxicam tablets in this study was higher and the geometric mean T_max(obs)_ and estimated plasma T_1/2(elim)_ were shorter when compared to previous studies examining the estimated pharmacokinetic parameters of meloxicam oral suspension following oral administration in non-lactating healthy goats at a lower oral dose of 0.5 mg/kg (Table 6) (2, 21). Since the breeds of goats used in two of the studies were similar (Alpines, Saanen, and Alpine X Saanen in this study and Saanen in Karademir et al., 2016) (21), the relatively minor differences in pharmacokinetics between these studies is most likely due to differences in product formulation (crushed tablets vs. suspension), route of administration (oral drench vs. ororuminal administration), dose (1 mg/kg vs. 0.5 mg/kg), and lactation status. For example, the shorter T_max(obs)_ reported in this study compared to studies using a commercially available oral suspension product (Table 6) could be due to the use of tablets dissolved in water, which could result in a more rapid absorption profile. From a regulatory standpoint, these results suggest that goats, similar to sheep (Table 6), eliminate meloxicam more rapidly than cattle following multiple-dose administration (28). Therefore, defaulting to cattle-based tissue WDIs or WDTs, depending on the approval status in the country of interest, when properly adjusted for dose and tolerance/MRL, would likely be sufficiently conservative from a human food safety point while still allowing for flexibility in extra-label drug use in goats. However, this study did not evaluate tissue residue depletion of meloxicam following multiple-dose administration in goats; therefore, further in silico modeling approaches (31) or tissue residue depletion studies are recommended to confirm this assumption.

**Table 6:** Comparison of non-compartmental pharmacokinetic parameters estimated from plasma meloxicam concentrations versus time data following oral administration of meloxicam between this study and previously published studies.

Parameter	Dairy goats (current study)	Goats (2)	Goats (21)	Dairy Goats (current study)	Dairy Cattle (27)	Sheep (28)
Dosing	1 mg/kg x 1	0.5 mg/kg x 1	0.5 mg/kg x 1	1 mg/kg every 24 h x 6	1 mg/kg every 24 h x 6	1 mg/kg every 24 h x 10
Number of animals	10	8	5	10	8	6
Breed(s)	Alpine, Saanen, Alpine x Saanen	Swedish Landrace	Saanen	Alpine, Saanen, Alpine x Saanen	Holstein	Unknown
Lactation Status	Early to Mid-Lactation	Non-Lactating	Non-Lactating	Early to Mid-Lactation	Mid-Lactation	Non-Lactating
Product Formulation	Crushed Tablets Suspended in Water	Commercial Oral Suspension Product	Commercial Oral Suspension Product	Crushed Tablets Suspended in Water	Crushed Tablets Suspended in Water	Crushed Tablets Suspended in Water
LOD (ng/mL)	4	Unknown, LOQ 0.5	10	4	5	4.9

Reporting Metric	Geometric Mean (range)	Mean (SD)	Mean (range)	Geometric Mean (range)	Geometric Mean (range)	Median (range)
$C_{\max(\text{obs})}$ (μg/mL)	1.28 (0.92–1.66)	0.736 (0.184)	0.71 (0.52–0.95)	1.59 (1.14–2.18)	1.5 (0.7–3.3)	1.65 (1.42–2.65)
$T_{\max(\text{obs})}$ (h)	7.46 (4–8)	15 (5)	14.33 (12–16)	5.66 (4–8)	----	4 (2–8)
$T_{1/2(\text{elim})}$ (h)	7.49 (5.45–14.37)	11.8 (1.7)	10.69 (8.60–12.17)	7.64 (5.61–9.47)	9.4 (5.6–12.3)	9.20 (6.90–10.46)
λz (1/h)	0.093 (0.048–0.13)	0.060 (0.009)	----	0.091 (0.07–0.12)	0.07 (0.06–0.12)	0.075 (0.066–0.10)
$\text{AU}C_{0-\infty }$ (h*μg/mL)	23.73 (16.20–33.91)	23.24 (7.33)	24.21 (16.15–34.71)	----	----	23.1 (18.9–26.8)
$\text{AU}C_{120-\infty }$ (h*μg/mL)	----	----	----	30.10 (19.66–49.87)	72.4 (22.1–297.8)	----

SD: standard deviation; LOQ: limit of quantification. The following individual parameter estimates are reported: C_max(obs)_: maximum observed plasma concentration; T_max(obs)_: time to maximum observed plasma concentration; T_½(elim)_: elimination half-life based on best fit data points; λz: elimination rate constant based on best fit data points; $({\text{AUC}}_{0-\infty ):}$ area under the plasma concentration vs. time curve from the time of the first dose extrapolated to infinity; $({\text{AUC}}_{120-\infty }):$ area under the plasma concentration vs. time curve from the time of the last dose extrapolated to infinity.

Early to mid-lactation goats in this study had a similar plasma geometric mean C_max(obs)_ but shorter geometric mean estimated plasma T_1/2(elim)_ compared to mid-lactation cattle (Table 6) (27) when administered the same oral dosing regimen of meloxicam at 1 mg/kg every 24 h for a total of 6 doses. Studies performed using single oral dose administration found that meloxicam has high oral bioavailability (96.5%) in goats (21) while cattle have slightly lower oral bioavailability at 87.2% (32). Meloxicam showed moderate accumulation in goats with a R_ac_ of 1.32 following repeated oral dosing at 1 mg/kg every 24 h for a total of 6 doses; however, this is less than that reported in beef calves (R_ac_ 2.1) (33). Therefore, it can be anticipated that the meloxicam withdrawal interval estimations reported here will need to be extended for cases using higher doses, more frequent administration, or for longer treatment courses. Since goats have higher oral bioavailability than cattle and lower accumulation index than calves, we speculate that the difference in half-life between the dairy goats in this study and the dairy cattle in Mzyk et al., 2023 (27) could be due to increased clearance in goats. However, since neither study had paired intravenous sampling data to determine bioavailability and true clearance, this assumption is purely speculative, and further research is needed.

Early to mid-lactation dairy goats in this study had a higher milk geometric mean C_max(obs)_ t shorter geometric mean estimated milk T_1/2(elim theo)_ and geometric mean estimated $\text{AU}C_{120-\infty (\text{theo})}$ when compared to mid-lactation dairy cattle (Table 7) (27) administered the same dosing regimen, suggesting increased milk clearance of meloxicam in goats compared to lactating cattle. Applying the FDA method to predict the milk WDI using the analytical LOD as the tolerance for 10 animals with triplicate milk samples (Scenario 1), a WDI of 70.1 h (rounded to 72 h based on 12 h milking intervals) was calculated. The terminal theoretical elimination half-life method using the estimated T_1/2(elim theo)_, which estimates a WDI based on theoretical 99% drug depletion, calculated a WDI (69.6 h rounded to 72 h) similar to the FDA method for Scenario 1, which is likely due to the very low LOD in this study (4 ng/mL) being close to theoretical 99% depletion. The EMA MRL method targeting the EMA MRL of 15 ng/mL produced the shortest WDI (64.97 h rounded to 72 h) among the methods used. However, based on a 12 h milking interval, this method ultimately would recommend the same WDI of 72 h despite the higher MRL (15 ng/mL). All of these estimated WDIs are shorter than the proposed 120 h WDI for healthy mid-lactation cattle targeting a higher LOD 5 ng/mL for the same dosing regimen (1 mg/kg PO every 24 h for a total of 6 doses) and milking frequency (12 h), indicating that meloxicam is more rapidly cleared from milk in healthy dairy goats than dairy cattle (27). In the absence of species-specific data, goats are often assumed to exhibit pharmacokinetic profiles similar to those of cattle, despite several studies, including the present one, indicating that goats may have equal or faster theoretical estimated drug elimination half-lives for milk. As such, milk WDIs or WDTs established for cattle may be reasonably applied to goats, provided they are appropriately adjusted to account for differences in route, dose, milking frequency, and analytical sensitivity, whether targeting the regulatory limit of detection (LOD), an established tolerance, or MRL. Based on the data in this study, from a food safety perspective, applying a cattle-derived milk WDI to dairy goats is likely to be conservative and offers an adequate margin of safety for population-based differences. However, this conservative approach may not be optimal for producers, as it could lead to longer WDIs than necessary. Still, when regulatory decisions rely on LOD-based tolerances using highly sensitive analytical methods, the conservatism of cattle WDIs could become less pronounced and thus be more acceptable for both human food safety and producer economics.

**Table 7:** Comparison of non-compartmental parameters estimated from concentration versus time data for meloxicam in milk following oral administration of meloxicam between this study and a previous study in dairy cattle (27).

Parameter	Dairy goats (current study)	Dairy cattle (27)
Dosing	1 mg/kg every 24 h for a total of 6 doses	1 mg/kg every 24 h for a total of 6 doses
Number of animals	10	8
Breed(s)	Alpine, Saanen, Alpine x Saanen	Holstein
Lactation status	Early to mid-lactation	Mid-lactation
Milking frequency	12 h	12 h
Product formulation	Crushed tablets suspended in water	Crushed tablets suspended in water
LOD (ng/mL)	4	5

Reporting metric	Geometric mean (range)	Geometric mean (range)
$C_{\max(\text{obs})}$ (μg/mL)	0.54 (0.26–1.0)	0.3 (0.2–0.5)
$T_{\max(\text{obs})}$ (h)	10 (10)	----
$T_{1/2(\text{elim theo})}$ (h)	6.96 (5.47–9.56)	9.6 (7.6–12.9)
$\text{AU}C_{120-\infty (\text{theo})}$ (h*μg/mL)	8.01 (3.65–17.21)	19.6 (7.4–66.5)
$\frac{\text{AU}C_{120-\infty \text{milk}\;(\text{theo})}}{\text{AU}C_{120-\infty (\text{plasma})}}$ (h*μg/mL)	0.27 (0.11–0.39)	0.3 (0.2–0.4)

LOD: limit of detection. The following individual parameter estimates are reported: Cmax(obs): maximum observed milk concentration; Tmax(obs): time to maximum observed milk concentration; T_½(elim theo)_: theoretical milk elimination half-life based on best fit data points; AUC_(120-∞ milk (theo))_: area under the theoretical milk concentration vs. time curve from the time of the last dose extrapolated to infinity; $\frac{{\text{AUC}}_{120-\infty \text{milk}\;(\text{theo})}}{{\text{AUC}}_{120-\infty (\text{plasma})}}:$ milk to plasma ratio (described by AUC_120-∞ milk(theo)/_ AUC_120-∞ plasma_) used to determine drug penetration into milk.

The milk to plasma ratio (described by AUC_120-∞ milk(theo)/_ AUC_120-∞ plasma_) is used to determine drug penetration into milk, with ratio values >1 indicating higher drug contents in milk when compared to plasma. For this study, the milk to plasma ratio was 0.27. This ratio is very similar to the milk to plasma ratio reported for single-dose IM or IV administration in goats (0.3) (23), which is slightly greater than the reported milk to plasma ratio for meloxicam following oral administration in cattle (Table 7) (27). However, for both goats and cattle, the drug penetration of meloxicam into milk is relatively low, and therefore, accumulation of meloxicam in milk over plasma is not expected in healthy animals. When comparing plasma vs. milk kinetics, the theoretical estimated milk T_1/2(elim theo)_ was very similar to the estimated plasma T_1/2(elim)_ following the final administered dose (Tables 3, 4), suggesting that plasma elimination roughly parallels milk elimination of meloxicam in goats. Therefore, plasma depletion of meloxicam can be used to guide milk depletion estimates in areas where milk testing is unavailable.

When applying the FDA method to predict milk WDI based on three different scenarios, the results for Scenario 1 (actual triplicate data) and Scenario 3 (simulating triplicate data from single replicate dataset) were very similar (70.1 vs. 70.3 h) and would produce the same milk WDI (72 h) when rounded to the next milking interval. These results suggest that when only single replicate data are available, it is adequate to use the Monte Carlo simulation technique to generate simulated data for two additional replicates to meet the data requirements of triplicate measurements in the FDA tolerance limit method for milk.

When comparing the calculated milk WDI between Scenario 1 and Scenario 2 (simulating 5 additional animals from a 5-animal dataset), there was a 12 h difference (i.e., 70.1 vs. 82.8 h) in estimated WDI, resulting in a slightly different WDI recommendation (72 h for Scenario 1 and 84 h for Scenario 2). The small difference between Scenario 1 and Scenario 2 can be attributed to the fourth terminal phase time point in the simulated five virtual goats. The base animals (Goats 1, 2, 3, 4, and 8) used to simulate the 5 additional animals were those that had measurable milk concentrations at the fourth terminal phase time point (mean concentration of 19.76 ng/mL and a coefficient of variation (CV) of 43%), which excludes the animals from the additional dataset that had milk concentrations below the LOD (4 ng/mL). By using only animals with measurable concentrations at the fourth terminal phase time point (47 h after the final administered dose), the WDI produced by Scenario 2 was biased towards the slower eliminating animals and therefore produced a more conservative WDI for drugs with more rapid milk residue depletion. In the present study, the difference was minimal, i.e., ~12 h. However, the exact difference will depend on the milk depletion profile of the specific drug in a particular species. Moreover, drugs that have prolonged or irregular estimated milk depletion kinetics may have more pronounced differences in WDI variability between actual data and simulated data approaches. These results suggest that, depending on the animal population, sampling scheme, and data selection, using the Monte Carlo simulation technique to generate additional virtual animal data introduces relatively more variability to the final predicted milk WDI when compared to simulating additional replicate data.

This study has a few limitations. While the study population included goats in a commercial dairy setting, only three breeds of dairy goats were represented in our study. Another potential limitation of this study is the use of a tablet formulation dissolved in water and administered orally as a drench, which may not fully replicate the pharmacokinetic profile of intact tablets or other commonly used formulations, such as oral suspensions or injectable products administered orally. Dissolving the tablet prior to administration could theoretically alter the rate or extent of absorption, potentially influencing the observed depletion profile. As such, caution should be exercised when extrapolating these findings to different dosing practices or formulations. This study also used only healthy goats, and while this is common practice for milk residue and WDI estimation studies, recent research has shown that the pharmacokinetics and milk residue depletion of meloxicam in dairy cattle is significantly altered by disease status (mastitis, post parturient hemoglobinuria) leading to prolonged detection of meloxicam residues in milk (27), which is similar to previous reports of altered milk clearance of flunixin in cattle with mastitis (34). While the regulatory approaches account for population variability through modeling the 95% confidence interval of the 99th or 95th percentile of the population, the WDI estimates from these methods are dependent on the population from which the data is sampled. Since this study only used healthy animals, we can presume that the regulatory methods used for WDI estimation will be most applicable to healthy animals and animals without severe systemic compromise. Since goats are commonly treated with meloxicam for pain or inflammation associated with disease, the WDIs estimated in this study may not be sufficient for animals with severe systemic disease or mastitis, and could result in violative residues and an increased food safety risk in these populations. Future studies could integrate physiologically based pharmacokinetic (PBPK) modeling to account for interindividual variability, refine dosing regimens, and enhance the accuracy of WDI estimations (31, 35, 36).

## 5 Conclusion

The findings from this study demonstrate that the theoretical estimated elimination half-life of meloxicam in milk is shorter for dairy goats than dairy cattle, resulting in shorter estimated WDIs for the same dosing regimen. Therefore, from a food safety perspective, cattle WDTs or estimated WDIs should be appropriately conservative for application to goats when properly adjusted for dose, route, and regulatory tolerances/MRLs. This study calculated a milk WDI of 72 h to target a tolerance/MRL of the study’s analytical limit of detection (4 ng/mL) following administration of meloxicam tablets (15 mg) dissolved in water and administered as an oral drench at 1 mg/kg every 24 h for 6 doses in lactating dairy goats. This recommendation is specific to the formulation and route used in this study and may not be directly applicable to other meloxicam formulations or administration methods. Practitioners should use professional judgment, and US practitioners should consult US FARAD or relevant regulatory guidance when considering extra-label drug use in dairy goats. The study also highlights the impact of data simulation approaches on milk WDI estimations, and that the Monte Carlo simulation of single replicate data may provide a reliable alternative when full triplicate datasets are unavailable. However, simulating additional virtual animals introduces slightly greater variability in WDI predictions, emphasizing the need for careful consideration of data selection criteria. While the estimated WDIs from this study provide valuable guidance for extra-label drug use in dairy goats, the results are based on healthy animals administered tablets dissolved in water as an oral drench, and potential alterations in formulation and drug disposition due to disease states warrant further investigation. Future research should focus on refining WDI recommendations by incorporating PBPK modeling and expanding studies to include diseased populations to enhance food safety outcomes and regulatory decision-making.

## Data Availability

The raw data supporting the conclusions of this article will be made available by the authors, without undue reservation.
